# Feasibility study of a field survey to measure antimicrobial usage in humans and animals in the Mekong Delta region of Vietnam

**DOI:** 10.1093/jacamr/dlab107

**Published:** 2021-08-12

**Authors:** Nguyen Van Cuong, Nguyen Phuong Cam Ly, Nguyen Thi Bich Van, Doan Hoang Phu, Bach Tuan Kiet, Vo Be Hien, Pawin Padungtod, Guy Thwaites, Marc Choisy, Juan Carrique-Mas

**Affiliations:** 1 Oxford University Clinical Research Unit, Ho Chi Minh City, Vietnam; 2 University of Alberta, Edmonton, Alberta, Canada; 3 Faculty of Animal Science and Veterinary Medicine, University of Agriculture and Forestry, Ho Chi Minh City, Vietnam; 4 Sub-Department of Animal Health and Production (SDAHP), Cao Lanh, Dong Thap, Vietnam; 5 Food and Agriculture Organization of the United Nations, Hanoi, Vietnam; 6 Centre for Tropical Medicine and Global Health, Nuffield Department of Medicine, Oxford University, Oxford, UK; 7 MIVEGEC, IRD, CNRS, University of Montpellier, Montpellier, France

## Abstract

**Background:**

Development of antimicrobial use (AMU) surveillance systems in humans and animals is a priority for many low- and middle-income countries; however accurate estimations are hampered by a diversity of animal production systems and metrics. The Mekong Delta region of Vietnam is a ‘hotspot’ of antimicrobial resistance and is home to a high density of humans and animal populations.

**Objectives:**

To measure and compare AMU using different metrics (standing population, biomass and population correction unit) in the Mekong Delta, and to explore the potential of field-based data collection methods in the design of AMU surveillance systems.

**Methods:**

We collected AMU data from humans and animals (chickens, ducks, Muscovy ducks, pigs) from 101 small-scale farms in the Mekong Delta over a fixed period (90 days in humans, 7 days in animals).

**Results:**

Humans used 7.1 DDD_kg_, or 175.9 mg of antimicrobial active ingredients (AAIs) per kg of standing body mass annually; animals consumed 60.9 ADD_kg_ or 1324 mg. In the Mekong Delta humans represented 79.3% of the total body mass but consumed 29.6% of AAIs by weight. AAIs regarded of critical importance by WHO represented 56.9% and 50.2% of doses consumed by animals and humans, respectively.

**Conclusions:**

Using a One Health approach, we show that AMU can potentially be estimated from cross-sectional surveys, although results are hypothetical due to small sample size and are sensitive to the chosen population denominator. The methodology proposed here can potentially be scaled up be applied to design AMU surveillance in low-resource settings, allowing AMU reduction efforts to be focused on particular animal species.

## Introduction

The global crisis of antimicrobial resistance (AMR) has a particularly severe impact on human and animal populations in low- and middle-income countries (LMICs) due to limited medical and veterinary care resources, high densities of farms and excessive levels of antimicrobial use (AMU).^1^^,^^2^ There is now considerable evidence of a link between excessive AMU and the occurrence of resistance in humans and animals.^3^^,^^4^

The Tripartite Global Action Plan, jointly developed by WHO, the Food and Agriculture Organization of the United Nations (FAO) and the World Organization for Animal Health (OIE)^5^ has established AMU surveillance as a key priority action. However, many LMICs have limited resources and capacity to collect AMU data. OIE compiles annual surveillance reports of AMU in animal production globally.^6^ However, these reports do not compare AMU between different animal species or production types, a necessary step in order to identify those production types where AMU/AMR is likely to be highest (i.e. hotspots). Furthermore, beyond limited research studies, there are no official global data on AMU in human community populations.^7^

Selection of appropriate metrics for AMU quantification is crucial since different metrics may result in different estimates.^8–10^ In its annual global reports, OIE relates quantities of antimicrobials (mg) to the weight of all animals produced, expressed as the sum of the bodyweight (kg) of slaughtered animals plus that of standing animals.^6^ The EU in its annual ESVAC report relates weight of antimicrobial active ingredients (AAIs) to animal populations computed using population correction units (PCU), which corresponds to the typical treatment weight for each of the standing and slaughtered animal species.^11^

A recent study estimated AMU in relation to animal and human biomass in Vietnam. The animal population denominator was calculated using a methodology analogous to the one adopted by OIE in its annual report.^12^ The study concluded that, overall, 262 mg and 247 mg of AAIs were used, respectively, per kg of animal and human biomass across the country. Overall, a total of 3838 t of antimicrobials were used in the country, of which 71.7% corresponded to animal and 28.3% to human AMU. However, such estimates were largely based on extrapolations from previous surveys on a limited number of species and locations. More accurate species-specific AMU information is required in order to identify production types where the selection pressure on AMR is greatest. The accurate estimation of AMU in human communities requires conducting longitudinal studies, which are both laborious and costly.

Located in the southwest of Vietnam, the Mekong Delta region (40 500 km^2^, 17.8 million population in 2019) is a hotspot of AMU/AMR in animal production. Previous studies conducted in this rural area have evidenced a high prevalence of AMR in commensal *Escherichia coli* from chickens, ducks and pigs.^13–15^ Similarly, non-typhoidal *Salmonella* and *Campylobacter* spp. from livestock and farmers also displayed resistance to a large number of antimicrobials.^16–18^ Unsurprisingly, the quantities of antimicrobials used in chicken and pig farms in the area are high.^9^^,^^19^ In addition to chickens and pigs, many small-scale farms are mixed and may include fish, ducks, Muscovy ducks and, to a lesser extent, ruminants (cattle and goats). The relative intensity of AMU in different animal species and humans has yet to be determined for the Mekong Delta and Vietnam as a whole.

Using a One Health approach, we quantified AMU in human residents and animals raised in smallholder farms in the Mekong Delta. The aims were: (i) to describe the types of antimicrobials used; (ii) to extrapolate gross quantities of antimicrobials to human and animal populations in the Mekong Delta of Vietnam; and (iii) to explore the feasibility of such survey-based methods to estimate AMU while highlighting potential differences depending on metric chosen. Results from this study may contribute to the design of country-level AMU surveillance systems.

## Materials and methods

### Ethics

Ethics approval for the study was obtained from the Oxford Tropical Research Ethics Committee (OxTREC), Oxford, UK (reference no. 533-19). Informed consent was obtained from all participants.

### Study design

We conducted a cross-sectional survey of poultry-raising households in 5 of the 12 districts of Dong Thap province (Mekong Delta, Vietnam) during July 2019. These districts were chosen based on convenience, all being located <30 km of the provincial capital. We requested from the chief officer of the District Veterinary Station (DVS) a list of small-scale poultry farms (i.e. raising chickens and/or ducks for commercial purposes) representative of their area. From each district, 20–25 were selected, and selected farm owners were invited to join the study. We aimed to sample ∼100 households. Visits were conducted by staff affiliated to the Sub-Department of Animal Health and Production of Dong Thap (SDAHP-DT) during July 2019. In each selected household, the main person identified as being responsible for taking care of the family members and animals was interviewed.

### Data collection

A structured questionnaire was developed to gather demographic data on human residents as well as food animals present in the farm on the visit date. To minimize recall bias, we enquired about consumption of AAIs over the latest 90 and 7 days for humans and animals, respectively. In all visits, the investigators requested to inspect the cabinets used to keep medicines of humans and animals. This was taken as an opportunity to discuss information on types, doses and concentration of any AAIs identified. All medicine products containing AAIs were singled out after reviewing the label or the prescription (for human medicines). AAI were classified based on the WHO list of antimicrobial agents.^20^ The concentration of AAIs contained in these products was described and was expressed in mg/tablet (human products) and mg or mL per kg of product (animal products). Those veterinary antimicrobials not appearing in the WHO list were classified according to the OIE list of antimicrobial agents.^21^

### Quantification of AMU in the surveyed population

We first estimated consumption using dose metrics [DDD kg (DDD_kg_) for humans, animal daily doses kg (ADD_kg_) for chickens, ducks, Muscovy ducks and pigs]. This was achieved by multiplying the reported number of days when antimicrobials were consumed over the observed period (90 and 7 days for humans and animals, respectively) of each treated person/flock/herd. These frequency estimates were also related to the ‘standing body mass’ of the surveyed population as well as to ‘body mass-time’ (i.e. ‘treatment intensity’). Estimates were aggregated for each species without performing farm adjustment. For example, consumption of ampicillin over 2 days (out of 90 days) by an 80 kg person equates to consumption of 160 DDD_kg_, or to 648.9 DDD_kg_ per year, or to 8.1 DDD_kg_ per kg of standing body mass. Such individual contributes to a body-mass time denominator of 365 × 80 = 29 200 (kg-days). The treatment intensity for each species was calculated as the ratio of the total number of doses to the kg-days.

For humans, the quantities of AAIs consumed were estimated from the actual doctors’ prescriptions where available. For animals, they were inferred from the preparation instructions of each product consumed. The estimated number of ADD_kg_ was multiplied by the ‘technical dose’ of each of the AAI consumed. The technical dose values for each AAI were inferred from the products’ preparation instructions (as written in the label) and from the prescription data in the case of human antimicrobial products. For animals, the technical ADD_kg_ was defined as 75% of the treatment daily dose for 1 kg of live animal bodyweight and was obtained from the preparation instructions as indicated in the products’ labels and consumption estimated from the weight (inferred from age) of the animal. Animal weights were derived from age based on previous studies.^9^^,^^22^^,^^23^ Respectively, 0.225 and 0.120 L were used as daily water intake and 0.063 kg and 0.037 kg as daily consumption of feed of 1 kg of poultry (any species) and pig.

To account for individuals that reported using medicine but did not remember whether it included AAI or not, we assumed that a fraction of them did actually consume an AAI. This was taken as the proportion consisting of an AAI among all medicine products. In cases where participants were sure of the consumption of AAIs, but did not remember for how many days, we assigned the average of the remaining observations. For individuals where AAI concentration or dosage data were missing, we extrapolated from similar products used in the same species.

### Hypothetical estimations of AMU in the Mekong Delta of Vietnam

AMU data by species obtained from the survey were extrapolated for human and animal populations in the Mekong Delta (2019) using four different metrics: (i) no. doses-kg (either DDD_kg_ or ADD_kg_) related to the total amount of kg-body mass-time for each species per year; (ii) weight of AMU related to standing body mass of human and animal populations per year; (iii) weight of AMU related to standing body mass of human and biomass of animal populations per year (i.e. the methodology used in the OIE report);^6^ (iv) weight of AMU related to standing body mass of human and PCU of animal populations per year (i.e. the ESVAC method).^24^

The human standing body mass was obtained by multiplying the number of individuals (from census data) by their bodyweight (from their age and gender).^25^^,^^26^ To estimate animal standing body mass, the number of animals of each species (from census data)^27^ was multiplied by the bodyweight of each species at their mid-age point.^12^ To estimate biomass of animals, the number of animals produced (from production data) of each species was multiplied by the total individual animal bodyweight at slaughter time. The number of PCUs for each animal species was calculated as the total number of animals multiplied by the average weight at treatment based on ESVAC guidelines.

## Results

### Participants and household farm characteristics

A total of 101 household farms with 316 human residents present in the farm household at the time of the visit were investigated. Each farm had a median of 3 (IQR 2–4) residents, with a median age of 38 (IQR 13–55) years. Over half (51.9%) were males. All interviewees were above 18 years old, and 82.2% were male. A total of 186 (58.8%) residents reported direct contact with animals in their farms (Table S1, available as Supplementary data at *JAC-AMR* Online).

Chickens were predominant (71.3% farms), followed by ducks (54.5%), pigs (19.8%) and Muscovy ducks (11.9%). Other species raised were fish (10.9%), cattle (5.9%), frogs (4.0%), goats (2.0%), geese and eels (1.0% each). Farms raising only one food animal species (42.6%) were predominant, followed by farms raising two (40.6%), three (11.9%), five (2.9%) and four (1.9%) species. The most common species combinations were ‘chicken-duck’ (17.8%), followed by ‘chicken-Muscovy duck’ (4.9%), ‘chicken-duck-pig’ (4.9%), ‘chicken-pig’ and ‘duck-pig’ (3.9% each). The median chicken flock age was 12 weeks (IQR 4–24) and the median flock size was 30 (IQR 15–75). The median duck flock age was 8 weeks (IQR 4–25), with a median size of 100 (IQR 40–700). The median pig age was 15 weeks (IQR 8–51), and the median herd size 10 (IQR 3–12) (Table S2).

### AMU in humans and animals in survey farms

A total of 173 participants (54.7%) reported using medicine over the last 90 days. However, only 121 participants (38.2%) kept doctors’ prescriptions. Forty-two out of 121 participants (34.7%) (median age 43 (IQR 7–55.5) years) confirmed using antimicrobial-containing products during the last 90 days. For the 52 participants who consumed medicine but did not remember whether it contained AAIs or not, we assumed that 34.7% did actually contain AAIs. Therefore antimicrobial consumption over the last 90 days was assumed for 60 (34.7%) individuals [42+(52 × 0.347)]. AMU was reported in a total of 109/284 (30.6%) flocks/herds (chicken, duck, Muscovy duck and pig) over the 7 days prior to the time of interview.

A total of 106 (32 in humans and 74 in animals) different AAI-containing products were identified. All human antimicrobial-containing products had been administered orally and contained one AAI. Fifty-four percent of antimicrobial-containing products administered to animals contained two AAIs. Most (75.6%) were administered orally, the remainder being injectable. A total of 9 and 63 antimicrobial-containing products were used in pig and poultry flocks, respectively. Twenty of the antimicrobial products administered to three animal species (chickens, ducks and pigs) were intended for human use. The technical daily doses of all AAI-containing products are given in Table S3 (Table 1). The average technical dose of human AAIs (DDD_kg_) was 17.3 mg/kg. For pigs and poultry the average technical daily dose (ADD_kg_) values were, respectively, 21.4 and 19.4 mg/kg for oral antimicrobials and 11.2 and 13.6 mg/kg for injectable ones.

**Table 1:** The technical dose (DDD_kg_ or ADD_kg_) corresponding to AAIs contained in AAI-containing products (32 intended for human use; 74 for animal use) reported in 101 study farms

Class	AAI	Humans (oral)		Poultry				Pigs			
				oral		injection		oral		injection	
		*n*	DDD_kg_ (±CV)	*n*	ADD_kg_ (±CV)	*n*	ADD_kg_ (±CV)	*n*	ADD_kg_ (±CV)	*n*	ADD_kg_ (±CV)
Tetracyclines^a^	tetracycline^c^	1	23.0 (±NC)	3	12.5 (±53.5)						
	oxytetracycline			9	6.9 (±87.8)	4	12.1 (±98.5)			1	3.7 (±NC)
	doxycycline			7	29.6 (±151.8)	1	8.4 (±NC)				
Sulfonamides	sulfaquinoxaline			2	18.3 (±76.6)						
	sulfamethoxazole^c^			1	18.7 (±NC)			1	40.5 (±NC)		
	sulfaguanidine^c^										
	sulfadimidine			2	63.4 (±66.1)						
	sulfadimethoxine			1	15 (±NC)						
Quinolones	norfloxacin			2	8.5 (±104.5)						
	marbofloxacin										
	enrofloxacin			6	11.0 (±36.3)	4	5.6 (±47.1)				
	ciprofloxacin^c^	1	18.4 (±NC)								
	ofloxacin	1	7.3 (±NC)								
Polypeptides^b^	colistin			11	3.1 (±36.3)			2	3.7 (±28.9)		
Penicillins^a^	ampicillin	1	23.0 (±NC)	5	8.1 (±82.6)			2	9.3 (±NC)		
	amoxicillin^c^	10	26.6 (±49.2)	3	13.7 (±28.3)	1	0.78 (±NC)	2	15.3 (±44.8)		
	penicillin V	1	23.0 (±NC)								
Macrolides^b^	tylosin			8	10.0 (±90.5)	2	9.3 (±28.2)			2	11.1 (±129.9)
	spiramycin	2	7.5 (±32.6)	1	0.45 (±NC)	1	3.0 (±NC)				
	erythromycin			1	15.0 (±NC)						
Lincosamides	lincomycin	1	23.0 (±NC)	1	0.75 (±NC)	3	5.1 (±39.7)				
Diaminopyrimidines	trimethoprim			3	3.6 (±51.9)			1	8.1 (±NC)		
First- and second-generation cephalosporins	cefuroxime	5	18.5 (±50.3)								
	cefotaxime^c^					1	9.7 (±NC)				
	cefalexin	4	13.8 (±44.5)	1	7.5 (±NC)						
	cefdinir	1	13.8 (±NC)								
Third-generation cephalosporins^b^	ceftiofur^c^					1	3.75 (±NC)				
	cefixime	1	9.2 (±NC)								
	cefpodoxime	2	3.2 (±60.6)								
	cefadroxil	1	9.2 (±NC)								
Amphenicols	thiamphenicol			1	24.7 (±NC)	4	16.8 (±55.9)			1	7.5 (±NC)
	florfenicol			2	5.6 (±120.3)	1	1.8 (±NC)			1	3.7 (±NC)
Aminoglycosides^a^	streptomycin			2	9.9 (±34.6)						
	spectinomycin			1	1.8 (±NC)	4	10.3 (±34.8)				
	kanamycin			2	8.0 (±75.6)						
	gentamicin			4	23.2 (±148.6)			2	5.5 (±14.4)		
Average all products		32	17.3 (±61.2)	52	12.4 (±156.4)	17	9.2 (±79.3)	5	11.9 (±99.1)	4	7.4 (±97.2)

AAIs used in animals intended for human use were excluded.

NC, not calculated; CV, coefficient of variation.

Critically important antimicrobial classes according to WHO are highlighted: ^a^high priority, ^b^highest priority; ^c^AAI used for animals but purchased from human medicine.

A total of 14 different AAIs belonging to 8 classes were consumed by humans. AAIs listed as of critical importance by WHO represented 50.2% of the total number of DDD_kg_. First- and second-generation cephalosporins were consumed the most (32.3% of total DDD_kg_), followed by penicillins (54.2%) and quinolones (9.5%). A total of 30 different AAIs belonging to 12 classes were consumed by animals. Critically important antimicrobials (CIAs) (WHO) represented 56.9% of the total number of doses given to animals (81.4% for chickens, 50.6% for ducks, 34.1% for Muscovy ducks and 61.8% for pigs). In terms of frequency, amphenicols (28.9% of ADD_kg_), tetracyclines (22.5%) and macrolides (18.6%) were the most consumed AAIs by chickens. In ducks, quinolones were the most used class (33.9%), followed by tetracyclines (21.0%) and penicillins (19.3%). In Muscovy ducks, quinolones represented 62.3% of doses, followed by macrolides (13.6%) and tetracyclines (12.0%). In pigs, penicillins (34.8%) represented the most frequently used class, followed by macrolides (28.7%) and amphenicols (10.4%) (Tables 2 and 3). Over 1 year, human residents in the farms surveyed were estimated to consume on average 7.1 DDD_kg_ or 175.9 mg of AAIs per kg of standing body mass. Of all animal species, Muscovy ducks consumed the most (196.1 ADD_kg_), followed by chickens (90.6 ADD_kg_), ducks (63.2 ADD_kg_) and pigs (22.5 ADD_kg_). However, in terms of mg of AAI per kg of standing body mass, chickens consumed the greatest amounts (3390.3 mg), followed by Muscovy ducks (3261.8 mg), ducks (1049.9 mg) and pigs (756.8 mg) (Table 4).

**Table 2:** Estimated annual AMU expressed in terms of doses per kg of bodyweight calculated from the small-scale farm survey

Class/AAI	Humans, DDD_kg_ (%)	Chickens, ADD_kg_ (%)	Ducks, ADD_kg_ (%)	Muscovy ducks, ADD_kg_ (%)	Pigs, ADD_kg_ (%)
Tetracyclines^a^					
tetracycline^c^	0.14 (2.0)	0.2 (0.3)	0.1 (0.1)	4.3 (2.1)	
oxytetracycline		14.4 (15.2)	6.9 (9.9)	15.6 (7.7)	0.4 (1.1)
doxycycline		14.5 (15.2)	4.2 (6.0)	0.3 (0.1)	
any	0.14 (2.0)	29.1 (30.7)	11.1 (16.0)	20.2 (9.9)	0.4 (1.1)
Sulphonamides					
sulfaquinoxaline		2.6 (2.7)			
sulfamethoxazole^c^		0.7 (0.7)			0.5 (1.4)
sulfaguanidine^c^					
sulfadimidine		0.8 (0.9)			
sulfadimethoxine		0.6 (0.6)			
any		4.7 (4.9)			0.5 (1.4)
Quinolones					
norfloxacin			0.1 (0.2)	131.6 (65.2)	
marbofloxacin		0.8 (0.8)			
enrofloxacin		5.4 (5.7)	28.0 (40.5)	3.2 (1.6)	3.1 (9.5)
ciprofloxacin^c^	0.49 (7.0)				
ofloxacin	0.16 (2.3)				
any	0.65 (9.3)	6.2 (6.5)	28.1 (40.7)	134.8 (66.8)	3.1 (9.5)
Polypeptides^b^					
colistin		6.3 (6.6)	12.0 (17.3)	27.0 (13.4)	5.6 (17.2)
any		6.3 (6.6)	12.0 (17.2)	27.0(13.4)	5.6 (17.2)
Penicillins^a^					
ampicillin	0.18 (2.6)	0.6 (0.6)	5.8 (8.4)	5.7 (2.8)	2.8 (8.6)
amoxicillin^c^	2.61 (37.2)	3.4 (3.5)	0.8 (1.2)		8.8 (26.9)
penicillin V	0.18 (2.6)				
any	2.97 (40.0)	4.0 (4.1)	6.6 (9.6)	5.7 (2.8)	11.6 (35.5)
Macrolides^b^					
tylosin		14.8 (15.5)	0.1 (0.2)	8.3 (4.1)	4.9 (15.0)
spiramycin	0.06 (0.91)	0.8 (0.8)	0.2 (0.2)		
erythromycin		2.6 (2.8)			
any	0.06 (0.91)	18.2 (19.1)	0.3 (0.4)	8.3 (4.1)	4.9 (15.0)
Lincosamides					
lincomycin	0.09 (1.34)	4.6 (4.8)	0.1 (0.2)		
any	0.09 (1.34)	4.6 (4.8)	0.1 (0.2)		
Diaminopyrimidines					
trimethoprim		0.4 (0.4)	0.1 (0.2)	5.7 (2.8)	0.5 (1.4)
any		0.4 (0.4)	0.2 (0.2)	5.7 (2.8)	0.5 (1.4)
First- and second-generation cephalosporins					
cefuroxime	1.21 (17.3)				
cefotaxime^c^			2.5 (3.6)		
cefalexin	1.36 (19.4)	0.2 (0.2)			
cefdinir	0.38 (5.5)				
any	2.95 (42.2)	0.2 (0.2)	2.5 (3.6)		
Third-generation cephalosporins^b^					
ceftiofur^c^			3.7 (5.4)		
cefixime	0.38 (5.5)				
cefpodoxime	0.08 (1.2)				
cefadroxil	0.03 (0.5)				
any	0.49 (7.2)		3.7 (5.4)		
Amphenicols					
thiamphenicol		11.2 (11.8)	0.6 (0.8)		0.4 (1.1)
florfenicol		0.6 (0.7)	0.1 (0.1)		2.4 (7.5)
any		11.8 (12.6)	0.7 (0.9)		2.8 (8.6)
Aminoglycosides^a^					
streptomycin		1.6 (1.7)	0.1 (0.1)		
spectinomycin		4.6 (4.8)	0.1 (0.2)		
kanamycin			0.1 (0.1)		
gentamicin		3.5 (3.7)	3.8 (5.4)	0.3 (0.1)	3.3 (10.2)
any		9.7 (10.2)	4.1 (5.8)	0.3 (0.1)	3.3 (10.2)
Grand total	7.0 (100)	95.2 (100)	69.0 (100)	202.1 (100)	32.7 (100)

Critically important antimicrobial classes according to WHO are highlighted: ^a^high priority, ^b^highest priority; ^c^AAIs in products administered to animal populations but intended for human use.

**Table 3:** Estimated annual AMU expressed in terms of weight of AAI as calculated from the farm survey

Class/AAI	Humans, mg (%)	Chickens, mg (%)	Ducks, mg (%)	Muscovy ducks, mg (%)	Pigs, mg (%)
Tetracyclines^a^					
tetracycline^c^	3.5 (2.0)	8.5 (0.3)	1.2 (0.1)	161.6 (5.0)	
oxytetracycline		249.6 (7.4)	91.5 (8.7)	156.8 (4.8)	2.8 (0.4)
doxycycline		500.1 (14.8)	128.3 (12.2)	72.7 (2.2)	
any	3.5 (2.0)	758.2 (22.5)	221.0 (21.0)	391.1 (12.0)	2.8 (0.4)
Sulphonamides					
sulfaquinoxaline		62.9 (1.9)			
sulfamethoxazole^c^		12.4 (0.4)			37.5 (1.0)
sulfaguanidine^c^					
sulfadimidine		156.5 (4.6)			
sulfadimethoxine		18.1 (0.5)			
any		249.9 (7.4)			37.5 (1.0)
Quinolones					
norfloxacin			0.3 (0.0)	1974.0 (60.5)	
marbofloxacin		5.9 (0.2)			
enrofloxacin		156.6 (4.6)	355.5 (33.9)	59.2 (1.8)	39.1 (5.2)
ciprofloxacin^c^	8.4 (4.8)				
ofloxacin	1.1 (0.6)				
any	9.5 (5.4)	162.5 (4.8)	355.8 (33.9)	2033.2 (62.3)	39.1 (5.2)
Polypeptides^b^					
colistin		58.9 (1.7)	92.8 (8.8)	222.2 (6.8)	62.7 (8.3)
any		58.9 (1.7)	92.8 (8.8)	222.2 (6.8)	62.7 (8.3)
Penicillins^a^					
ampicillin	4.5 (2.5)	13.6 (0.4)	160.6 (15.3)	53.8 (1.6)	78.7 (10.4)
amoxicillin^c^	86.5(49.2)	243.1 (7.2)	42.0 (4.0)		184.4 (24.4)
penicillin V	4.5 (2.5)				
any	95.5 (54.2)	256.7 (7.6)	202.6 (19.3)	53.8 (1.6)	263.1 (34.8)
Macrolides^b^					
tylosin		382.8 (11.3)	5.6 (0.5)	443.1 (13.6)	217.5 (28.7)
spiramycin	0.5 (0.3)	10.7 (0.3)	0.2 (0.0)		
erythromycin		236.6 (7.0)			
any	0.5 (0.3)	630.1 (18.6)	5.8 (0.5)	443.1 (13.6)	217.5 (28.7)
Lincosamides					
lincomycin	2.0 (1.1)	69.3 (2.0)	1.6 (0.2)		
any	2.0 (1.1)	69.3 (2.0)	1.6 (0.2)		
Diaminopyrimidines					
trimethoprim		4.5 (0.1)	0.8 (0.1)	76.9 (2.4)	7.5 (1.0)
any		4.5 (0.1)	0.8 (0.1)	76.9 (2.4)	7.5 (1.0)
First- and second-generation cephalosporins					
cefuroxime	25.8 (14.6)				
cefotaxime^c^			24.3 (2.3)		
cefalexin	29.8 (16.9)	6.9 (0.2)			
cefdinir	1.5 (0.8)				
any	57.1 (32.3)	6.9 (0.2)	24.3 (2.3)		
Third-generation cephalosporins^b^					
ceftiofur^c^			42.1 (4.0)		
cefixime	5.6 (3.2)				
cefpodoxime	0.7 (0.4)				
cefadroxil	1.0 (0.5)				
any	7.3 (4.1)		42.1 (4.0)		
Amphenicols					
thiamphenicol		976.9 (28.8)	27.7 (2.6)		5.5 (0.7)
florfenicol		2.1 (0.1)	0.5 (0.0)		73.2 (9.7)
any		979.0 (28.9)	28.2 (2.6)		78.7 (10.4)
Aminoglycosides^a^					
streptomycin		25.5 (0.0)	1.4 (0.1)		
spectinomycin		140.9 (4.2)	3.2 (0.3)		
kanamycin		1.6 (0.1)	0.7 (0.1)		
gentamicin		46.2 (1.4)	69.7 (6.6)	41.6 (1.3)	47.9 (6.3)
any		214.2 (6.4)	75.0 (7.1)	41.6 (1.3)	47.9 (6.3)
Grand total	175.8 (100)	3390 (100)	1050 (100)	3261 (100)	756.8 (100)

Critically important antimicrobial classes according to WHO are highlighted: ^a^high priority, ^b^highest priority; ^c^AAIs in products administered to animal populations but intended for human use.

**Table 4:** Estimation of AMU in animals and humans from a survey of 101 farming households

	Humans	Chickens	Ducks	Muscovy ducks	Pigs	Total
No. households	101	72	55	12	20	101
No. individuals/animals	316	15 881	42 256	1308	494	–
Total standing body mass (kg)	14 420	16 717	88 047	820	21 145	141 149
Total kg-days per year	5 263 300	6 101 851	32 137 480	299 236	7 717 925	51 519 792
Gross AMU per year						
no. daily doses kg	101 909	1 514 730	5 573 085	160 845	476 168	7 826 737
no. mg AAIs	2 535 937	56 677 971	92 445 007	2 674 141	16 003 475	170 336 531
AMU related to population						
no. daily doses per kg standing kg	7.1	90.6	63.3	196.2	22.5	55.4
no. mg AAIs per kg body mass	175.9	3390.4	1050.0	3261.1	756.8	1206.8
treatment intensity (per 1000 days)	19.3	248.2	173.4	537.5	61.6	151.9

**Figure 1.**
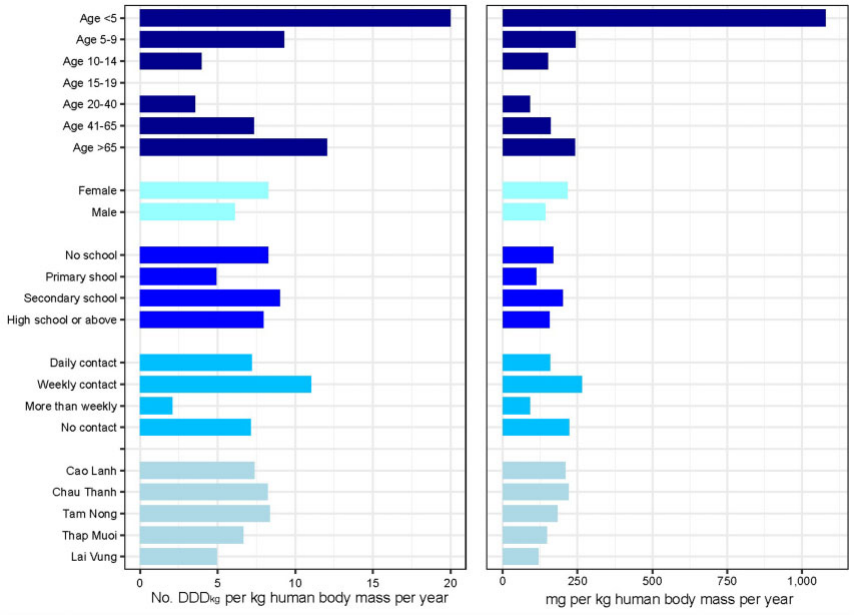
AMU in human participants by their sociodemographic characteristics.

**Figure 2.**
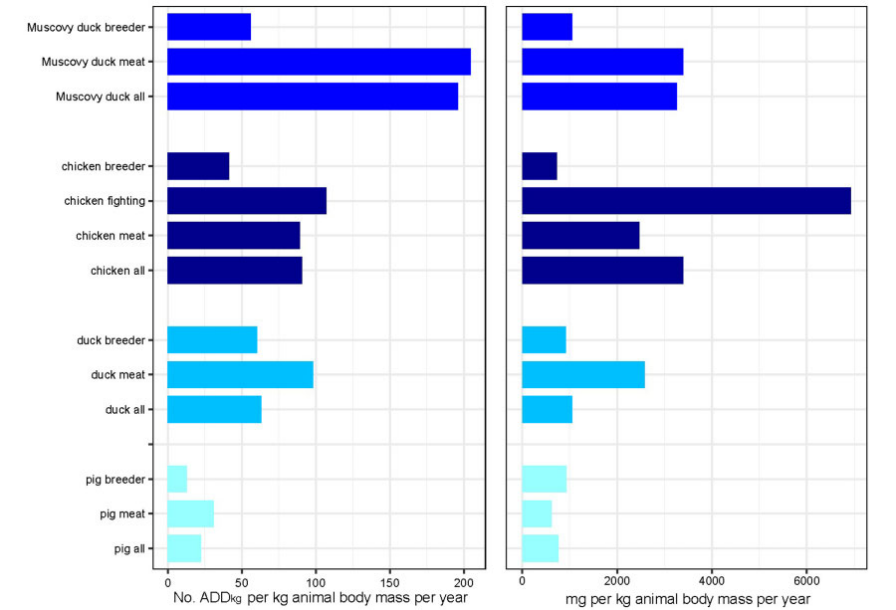
AMU consumption in animal by four species with specific type of production.

Human individuals aged less than 5 and more than 65 years consumed considerably more antimicrobials than people in other age categories (Figure 1). There were no noticeable differences in antimicrobial consumption with respect to other sociodemographic factors. AMU in the animal species stratified by production purpose is displayed in Figure 2.

In general, animals raised for meat consumed more antimicrobials than those raised for breeding purposes. In terms of quantities, fighting chickens were the target of highest levels of AMU. Detailed data on AMU of individuals and animal groups of each species at farm level is provided in Table S4.

### Hypothetical estimates of AMU for the Mekong Delta region

Calculations of animal and human standing body mass, biomass and weight at treatment are presented in Tables S5 and S6. The hypothetical estimated annual amounts of antimicrobials consumed by each species (including humans) related to standing body mass, biomass and PCU for the Mekong Delta region are graphically displayed in Table 5. Calculations are shown in Table S7.

**Table 5:** Annual antimicrobial consumption related to standing body mass by species in the Mekong Delta of Vietnam

	Humans	Chickens	Ducks	Muscovy ducks	Pigs	Other species	Total (A)	Total (B)
Standing body mass (kg)	792 428 735 (79.3%)	58 373 100 (5.8%)	38 699 000 (3.9%)	3 844 800 (0.4%)	106 092 304 (10.6%)	103 348 407 (NC)	1 102 786 346	999 437 939 (100%)
Sampling fraction	1/54 953	1/3492	1/439	1/4689	1/5017	NC	NC	NC
Biomass (kg)	792 428 735 (58.4%)	155 491 200 (11.5%)	114 916 000 (8.5%)	12 742 400 (0.9%)	281 462 894 (20.7%)	91 828 583 (NC)	1 448 869 812	1 357 041 229 (100%)
PCU (kg)	792 428 735 (66.7%)	86 384 000 (7.3%)	63 203 800 (5.3%)	6 769 400 (0.6%)	241 004 200 (20.3%)	81 929 458	1 271 719 593	1 189 790 135 (100%)
No. kg of AAI	139 357 (29.6%)	197 910 (42.0%)	40 630 (8.6%)	12 538 (2.7%)	80 289 (17.1%)	NC	NC	470 724 (100%)
No. doses kg of AAIs (%)	4 633 560 725 (31.3%)	4 169 821 320 (28.1%)	2 932 476 200 (19.8%)	680 938 560 (4.6%)	2 405 441 499 (16.2%)	NC	NC	14 822 238 304 (100%)
No. mg AAIs per kg biomass	175.9	1272.8	353.6	983.9	285.2	NC	NC	346.9
No. mg AAIs per kg PCU	175.9	2291.0	642.8	1852.1	333.14	NC	NC	395.6

NC, not calculated.

Total (A): all terrestrial animal species included.

Total (B): excludes animal species other than pigs, chickens, ducks and Muscovy ducks.

A total of 14.8 billion doses-kg, or 470.7 tonnes of AAIs were hypothetically used in the Mekong Delta by humans, pigs, chickens, ducks and Muscovy ducks combined. Considering only pigs, chickens, ducks and Muscovy ducks combined, animals represented 41.6%, 33.3% and 20.1% of total biomass, PCU and standing body mass, respectively. However, in terms of doses, animal consumption of the four animal species represented 69.7% of total AMU measured (71.4% in terms of quantity). Depending on whether AMU was related to total standing bodyweight, biomass or PCU, a total of 1600 mg, 586 mg or 833 mg were consumed per kg. Chickens were the target of 42% of the total volume of antimicrobials (28.1% of the doses). In terms of total number of doses consumed, ducks were second (19.8%), although third in terms of quantity (8.6%) (pigs were second with 16.2%).

## Discussion

Quantification of AMU through surveillance in human and animal systems has been set by international agencies as a priority in order to successfully tackle the global threat of AMR.^5^^,^^28^ Although AMU surveillance systems have been established in a number of developed countries (notably in the EU), these are only now starting to emerge in LMICs. Most of the existing surveillance systems are based on sales data and face the difficulty of assigning species to antimicrobial sales, since antimicrobial formulations often can be theoretically used on several species. By conducting AMU surveys at the end-user level, we are able to overcome this limitation. We advocate for using a random sampling technique, using the list of households and farms as a sampling frame. Collection of data on AMU in all relevant animal species would be important in any meaningful surveillance system, since species other than pigs, ducks, Muscovy ducks and chickens represent about 33% of the standing animal bodyweight in the Mekong Delta.

Our study had two major limitations that may compromise the observed results: (i) it was based on a survey of a small number of household farms and was not based on a true random sample; and (ii) it was conducted during a fixed period (July). In the Mekong Delta region, there is increased disease in livestock during June–November (the rainy season) (J. Carrique-Mas, unpublished data) with a potential concomitant increase in AMU. These limitations will inevitably compromise the validity of results. In any case, we should exercise extreme caution in interpreting them. Future studies should be based on a true random sample, there should be a sample size calculation for each animal species separately in order to reduce the standard error, and the sampling period should be spread over the year.

In our study, humans used 19.3 DDDs per 1000 inhabitants per day. These estimates are lower than a previous estimate for Vietnam (∼32 DDDs), but in line with the 2019 EU average (20.1 DDDs, with a country range of 9.7–34.0).^11^ A 2018 report shows that AMU in humans in Thailand was 74.4 DDDs.^29^ It is possible that some of the observed differences are due to our study not including AMU in hospitals/human healthcare settings. In animals, however, our data (833 mg AAIs per kg PCU) were similar to recent surveillance data from Thailand (711 mg/PCU in 2018),^29^ but much higher than in the EU (country mean 103.2 mg/PCU).^30^ Our data also indicate higher AMU in animals compared with OIE global estimates (586 mg compared with 240.5 mg AAI/kg biomass).

Our animal AMU findings in terms of biomass (346 mg/kg) were marginally higher than a previous estimate for the country as a whole (261.7 mg/kg). In contrast, our findings on AMU in humans were slightly lower (175.9 mg/kg versus 261.7 mg/kg).^12^

There are no available data on AMU in species other than chickens in the Mekong Delta. In this study, chickens consumed ∼245 doses per 1000 days, about a third less than amounts measured in a previous study in the area (382 per 1000 days).^9^ However, in terms of volume (quantity), this species consumed disproportionately high amounts of antimicrobials, driven by AMU in adult fighting cockerels, which represented a high fraction of the chicken farms (11/21) and consumed greater amounts of antimicrobials. AMU in Muscovy ducks was disproportionately high in terms of treatment intensity; however in terms of weight their consumption was similar to chickens. These differences are partly explained by the different ages of the animals included in the sample as well as differences in strength of antimicrobials used.

The results highlighted that the intensity of AMU in each animal species highly depended on the metrics used. Estimation of AMU using different metrics should allow comparisons with other studies. The magnitude of AMU appears highest when the weight of AAIs is expressed in relation to animal standing body mass, followed by PCU and biomass. We believe that AMU related to standing biomass should allow a fair comparison between animals and humans, since, at a broader geographical level, the species’ body mass is not likely to change substantially over time.

We found a greater diversity of AAIs used in animals (30 AAIs, belonging to 12 classes) compared with humans (14 AAIs, belonging to 8 classes). The antimicrobial classes used by animals in this study were very similar to a previous study in the same area.^9^ Use of CIAs was higher in animals (56.9% of all use) than in humans (50.2%), although the CIA classes were different among human and animals species (penicillins and third-generation cephalosporins in humans; tetracyclines, penicillins and polypeptides in animals).

Using a relatively simple One Health survey design, we were able to measure AMU in the four major animal species in the Mekong Delta of Vietnam and compare it to that of humans in rural community settings in the same area using different metrics. Although the estimates are hypothetical and subject to uncertainty given the pilot study design, we believe that this data collection methodology is feasible. Furthermore, our approach is affordable and therefore appropriate for LMICs. If performed at a sufficiently large scale, such studies could be used to establish national AMU estimates for different animal species. Human AMU data should be complemented with data on AMU in hospital settings.

## Supplementary Material

dlab107_Supplementary_Data
