# Supplementary material for: Feasibility study of a field survey to measure antimicrobial usage in humans and animals in the Mekong Delta region of Vietnam
Source: JAC Antimicrob Resist. 2021 Aug 12;3(3):dlab107. doi: 10.1093/jacamr/dlab107 (PMC8360299; doi:10.1093/jacamr/dlab107)
Supplement: dlab107_Supplementary_Data [file dlab107_supplementary_data.zip › Tables_S1_S2_S5_S6_S7.docx]

**Table S1.** Socio-demographic characteristics of 316 residents of 101 small-scale farming households.

|  | Interviewee  N=101 (%) | Other participants N=215 (%) | All participants N=316 (%) |
| --- | --- | --- | --- |
| District |  |  |  |
| *Cao Lanh* | 21 (20.8) | 50 (23.3) | 71 (22.5) |
| *Chau Thanh* | 20 (19.8) | 45 (20.9) | 65 (20.6) |
| *Lai Vung* | 21 (20.8) | 39 (18.1) | 60 (19) |
| *Thap Muoi* | 20 (19.8) | 53 (24.7) | 73 (23.1) |
| *Tam Nong* | 19 (18.8) | 28 (13) | 47 (14.9) |
| Age |  |  |  |
| *<5* | - | 31 (14.4) | 31 (9.8) |
| *5-9* | - | 29 (13.4) | 29 (13.4) |
| *10-14* | - | 25 (11.6) | 25 (11.6) |
| *15-19* | - | 11 (5.1) | 11 (5.1) |
| *20-40* | 27 (26.7) | 43 (20) | 70 (22.2) |
| *41-65* | 56 (55.4) | 48 (22.3) | 104 (32.9) |
| *>65* | 18 (17.8) | 28 (13) | 46 (14.6) |
| Gender |  |  |  |
| *Male* | 83 (82.2) | 81 (37.7) | 164 (51.9) |
| *Female* | 18 (17.8) | 134 (62.3) | 152 (48.1) |
| Frequency of contact with animals |  |  |  |
| *No contact* | 2 (2) | 112 (52.0) | 114 (36.0) |
| *Daily* | 96 (95) | 77 (35.8) | 173 (54.7) |
| *Weekly* | 1 (1) | 12 (5.6) | 13 (4.1) |
| *More often* | 2 (2) | 14 (6.5) | 16 (5.0) |
|  |  |  |  |
| Education achivement* |  |  |  |
| *No school* | 5 (5) | 20 (9.3) | 25 (7.9) |
| *Primary school* | 41 (40.6) | 48 (22.3) | 89 (28.2) |
| *Secondary school* | 33 (32.7) | 36 (16.7) | 69 (21.8) |
| *High school or higher* | 22 (21.8) | 15 (7) | 37 (11.7) |

*Applicable to residents aged >18 years.

**Table S2.** Species and production purpose of animals raised in 101 small-scale farming households.

| Species | Farming type | No. farms  (N=101) (%) | Age (weeks) (median) [IQR] | Flock size (median) [IQR] |
| --- | --- | --- | --- | --- |
| Chicken |  | 72 (71.3) | 12 [4-24] | 30 [15-70] |
|  | *Meat* | 53 (52.5) | 10.5 [4-14] | 40 [20-200] |
|  | *Fighting* | 22 (21.8) | 12 [4-27] | 30 [20-50] |
|  | *Breeding/layer* | 21 (20.7) | 48 [30-66] | 10 [6-23] |
| Duck |  | 55 (54.5) | 8 [4-25] | 100 [40-700] |
|  | *Meat* | 37 (36.6) | 6 [4-9] | 80 [37-182] |
|  | *Breeding/layer* | 21 (20.8) | 28 [24-46] | 1,500 [500-2,300] |
| Pig |  | 20 (19.8) | 15 [8-51] | 10 [3-12] |
|  | *Meat* | 17 (16.8) | 12 [8-20] | 10 [7-12] |
|  | *Breeding* | 6 (5.9) | 48 [32-96] | 3 [2-10] |
| Muscovy duck | | 12 (11.9) | 4 [2-12] | 30 [10-77] |
|  | *Meat* | 11 (10.9) | 4 [2-8] | 40 [13-100] |
|  | *Breeding/layer* | 3 (3) | 52 [32-81] | 4 [3-11] |
| Fish |  | 11 (10.9) | 14 [12-19] | 1,000 [162-1,000] |
|  | *Meat* | 9 (8.9) | 14 [12-17] | 1,000 [120-1,000] |
|  | *Breeding* | 2 (2) | 19 [13-25] | NA |
| Cattle |  | 6 (5.9) | 80 [52-240] | 2 [1-2] |
|  | *Meat* | 5 (5.0) | 56 [40-92] | 2 [1-2] |
|  | *Breeding* | 2 (2.0) | 276 [258-294] | 1 [NC] |
| Frog |  | 4 (4.0) | 5 [3-8] | 15,000 [15,000-55,000] |
|  | *Meat* | 4 (4.0) | 4 [3-5] | 22,500 [15,000-67,500] |
|  | *Breeding* | 1 (1.0) | 32 [32-32] | 500 [NC] |
| Goat |  | 2 (2.0) | 32 [26-40] | 5 [NC] |
|  | *Meat* | 1 (1.0) | 20 [20-20] | 5 [NC] |
|  | *Breeding* | 1 (2.0) | 40 [36-44] | 5 [NC] |
| Geese | *Breeding/layer* | 2 (2.0) | 67 [48-85] | 2 [NC] |

NC: Not calculated; NA: Not available.

**Table S5**. Standing bodymass of humans living in the Mekong Delta region of Vietnam. It was estimated using age-gender-weight metrics from census (1) and published data (2).

| Age (years) | Total population (1) | | | | Average weight (kg) (2) | | | | Estimated bodymass (kg) | |
| --- | --- | --- | --- | --- | --- | --- | --- | --- | --- | --- |
|  | Males | Females | Total | Males | | Females | Males | Females | | Total |
| 0 to 4 | 779,689 | 677,961 | 1,457,650 | 8.0 | | 7.8 | 6,237,511 | 5,288,093 | | 11,525,605 |
| 5 to 9 | 743,489 | 646,657 | 1,390,146 | 14.7 | | 14.7 | 10,929,295 | 9,505,858 | | 20,435,153 |
| 10 to 14 | 684,944 | 603,585 | 1,288,529 | 25.6 | | 27 | 17,534,568 | 16,296,790 | | 33,831,357 |
| 15 to 19 | 625,648 | 570,054 | 1,195,702 | 41.4 | | 35.4 | 25,901,830 | 20,179,916 | | 46,081,745 |
| 20 to 40 | 3,029,801 | 2,852,915 | 5,882,716 | 58.4 | | 50.8 | 176,940,398 | 144,928,062 | | 321,868,460 |
| 41 to 65 | 2,611,166 | 2,637,830 | 5,248,996 | 58.4 | | 50.8 | 152,492,079 | 134,001,773 | | 286,493,852 |
| > 65 | 535,755 | 805,206 | 1,340,961 | 58.4 | | 50.8 | 31,288,117 | 40,904,446 | | 72,192,563 |
| Total | 9,010,493 | 8,794,207 | 17,804,700 | - | | - | 421,323,798 | 371,104,937 | | 792,428,735 |

(1) Population pyramid of Vietnam, 2019. 2019 (<https://www.populationpyramid.net/vietnam/2019>); (2) Carrique-Mas, J.J., *et al*., An estimation of total antimicrobial usage in humans and animals in Vietnam. Antimicrob Resist Infect Control, 2020. **9**: p. 16.

**Table S6.** Estimation of animal ‘standing bodymass’, animal ‘biomass’ and animal ‘PCU’. It was estimated from number of animal (from census (1), production data (2) and weight of animals, either from their mid-age weight (3), slaughter weight (based on OIE) (4) or treatment weight (based on ESVAC) (5). Mid-point weight of meat animal equals 50% slaughter weight.

| Species | Production type | No. animals | | Weight of animals | | | Total animal weight | | |
| --- | --- | --- | --- | --- | --- | --- | --- | --- | --- |
|  |  | Standing population (census data) (1) | No. slaughtered animals (production data) (2) | Mid-point weight (kg) (3) | Slaughter weight (kg) (4) | Treatment weight (kg) (5) | Standing bodymass (kg)  (1)*(3) | Biomass (kg)  (2*4) | PCU (kg)  (2*5) |
| Pig | Meat | 1,487,452 | 2,974,904 | 39.3 | 78.6 | 65.0 | 58,456,864 | 233,827,454 | 193,368,760 |
|  | Breeder | 198,481 | 198,481 | 240.0 | 240.0 | 240.0 | 47,635,440 | 47,635,440 | 47,635,440 |
| Chicken | Meat | 40,853,000 | 74,381,000 | 0.9 | 1.8 | 1.0 | 36,767,700 | 133,885,800 | 74,381,000 |
|  | Breeder | 12,003,000 | 12,003,000 | 1.8 | 1.8 | 1.0 | 21,605,400 | 21,605,400 | 12,003,000 |
| Duck | Meat | 15,923,000 | 46,070,000 | 1.0 | 2.0 | 1.1 | 15,923,000 | 92,140,000 | 50,677,000 |
|  | Breeder | 11,388,000 | 11,388,000 | 2.0 | 2.0 | 1.1 | 22,776,000 | 22,776,000 | 12,526,800 |
| Muscovy duck | Meat | 1,959,000 | 3,760,000 | 1.6 | 3.2 | 1.7 | 3,134,400 | 12,032,000 | 6,392,000 |
|  | Breeder | 222,000 | 222,000 | 3.2 | 3.2 | 1.7 | 710,400 | 710,400 | 377,400 |
| Quail | Meat | 2,871,900 | 6,980,000 | 0.07 | 0.13 | 0.08 | 186,674 | 907,400 | 558,400 |
|  | Breeder | 319,100 | 319,100 | 0.13 | 0.13 | 0.08 | 41,483 | 41,483 | 25,528 |
| Bovine | Meat | 808,955 | 292,386 | 75.0 | 150.0 | 140.0 | 60,671,625 | 43,857,900 | 40,934,040 |
|  | Breeder | 70,707 | 70,707 | 300.0 | 300.0 | 425.0 | 21,212,100 | 21,212,100 | 30,050,475 |
| Buffalo | Meat | 20,736 | 7,053 | 150.0 | 300.0 | 140.0 | 3,110,400 | 2,115,900 | 987,420 |
|  | Breeder | 2,303 | 2,303 | 500.0 | 500.0 | 425.0 | 1,151,500 | 1,151,500 | 978,775 |
| Goat | Meat | 358,592 | 250,724 | 37.5 | 75.0 | 20.0 | 13,447,200 | 18,804,300 | 5,014,480 |
|  | Breeder | 39,843 | 39,843 | 75.0 | 75.0 | 75.0 | 2,988,225 | 2,988,225 | 2,988,225 |
| Sheep | Meat | 838 | 560 | 37.5 | 75.0 | 20.0 | 31,425 | 42,000 | 11,200 |
|  | Breeder | 93 | 93 | 75.0 | 75.0 | 75.0 | 6,975 | 6,975 | 6,975 |
| Goose | Meat | 203,000 | 164,000 | 1.6 | 3.2 | 1.7 | 324,800 | 524,800 | 280,440 |
|  | Breeder | 55,000 | 55,000 | 3.2 | 3.2 | 1.7 | 176,000 | 176,000 | 93,500 |

(1) (2) [Thống kê chăn nuôi Việt Nam 01/01/2020](https://channuoivietnam.com/?wpdmact=process&did=MjMxOS5ob3RsaW5r). Available at: <https://channuoivietnam.com/thong-ke-chan-nuoi/>

(4) OIE Annual Report on Antimicrobial Agents Intended for Use in Animals: Methods Used. Available at: <https://www.frontiersin.org/files/Articles/462898/fvets-06-00317-HTML/image_m/fvets-06-00317-t002.jpg>

(5) European Surveillance of Veterinary Antimicrobial Consumption (ESVAC) Sales Data and Animal Population Data Collection Protocol. Available at: <https://www.ema.europa.eu/en/documents/other/european-surveillance-veterinary-antimicrobial-consumption-esvac-web-based-sales-animal-population_en.pdf> ; *Pig production data were estimated from census data.

**Table S7**. Calculation of total AMU in animals and humans in the Mekong Delta region of Vietnam

|  | | Pigs | | | Chickens | | | Ducks | | | Muscovy ducks | | | Humans |
| --- | --- | --- | --- | --- | --- | --- | --- | --- | --- | --- | --- | --- | --- | --- |
|  |  | Meat | Breeder | All | Meat | Breeder | All | Meat | Breeder | All | Meat | Breeder | All |  |
| Survey | Standing bodymass (kg) | 9,726 | 11,419 | 21,415 | 11,976 | 985 | 12,961 | 7,262 | 85,018 | 92,280 | 781 | 46 | 827 | 14,420 |
|  | mg AAI/kg standing bodymass | 611.8 | 927 | 756.8 | 2471 | 728.5 | 3,390 | 2,574.70 | 913.7 | 1,049.90 | 3394.5 | 1047.6 | 3,261.80 | 175.8 |
|  | No. DDDkg/ kg standing bodymass | 30.8 | 12.7 | 22.5 | 89.2 | 41.2 | 90.6 | 98.2 | 60.1 | 63.2 | 204.6 | 55.8 | 196.1 | 7.0 |
|  | Treatment intensity (No. doses per 1,000 days) | 84.3 | 34.8 | 61.6 | 244.4 | 113.0 | 248.2 | 269.4 | 164.7 | 173.4 | 560.8 | 153.8 | 537.5 | 19.3 |
| Mekong Delta | Standing bodymass (kg) | 58,456,864 | 47,635,440 | 106,092,304 | 36,767,700 | 21,605,400 | 58,373,100 | 15,923,000 | 22,776,000 | 38,699,000 | 3,134,400 | 710,400 | 3,844,800 | 792,428,735 |
|  | Bodymass-days (kg-days) | 21,336,755,214 | 17,386,935,600 | 38,723,690,814 | 13,420,210,500 | 7,885,971,000 | 21,306,181,500 | 5,811,895,000 | 8,313,240,000 | 14,125,135,000 | 1,144,056,000 | 259,296,000 | 1,403,352,000 | 289,236,488,275 |
|  | Biomass (kg) | 233,827,454 | 47,635,440 | 281,462,894 | 133,885,800 | 21,605,400 | 155,491,200 | 92,140,000 | 22,776,000 | 114,916,000 | 12,032,000 | 710,400 | 12,742,400 | 792,428,735 |
|  | PCU (kg) | 193,368,760 | 47,635,440 | 241,004,200 | 74,381,000 | 12,003,000 | 86,384,000 | 50,677,000 | 12,526,800 | 63,203,800 | 6,392,000 | 377,400 | 6,769,400 | 792,428,735 |
|  | No. DDDkg | 1,800,471,411 | 604,970,088 | 2,405,441,499 | 3,279,678,840 | 890,142,480 | 4,169,821,320 | 1,563,638,600 | 1,368,837,600 | 2,932,476,200 | 641,298,240 | 39,640,320 | 680,938,560 | 4,633,560,725 |
|  | mg AAI | 35,763,909,395 | 44,158,052,880 | 79,921,962,275 | 90,852,986,700 | 15,739,533,900 | 106,592,520,600 | 40,996,948,100 | 20,810,431,200 | 61,807,379,300 | 10,639,720,800 | 744,215,040 | 11,383,935,840 | 114,549,627,433 |
